# Supplementary material for: Prevalence of pregnancy termination and associated factors among married women in Papua New Guinea: A nationally representative cross-sectional survey
Source: PLoS One. 2024 Sep 5;19(9):e0309913. doi: 10.1371/journal.pone.0309913 (PMC11376535; doi:10.1371/journal.pone.0309913)
Supplement: Attachment — Submitted filename: comments for author.docx [file pone.0309913.s002.docx]

Thank you dear editor for inviting me to review this manuscript which provides interesting findings for policy makers in the respective country. I have provided the following comments for the author.

1.Prevalence of pregnancy termination and associated factors among married women in Papua New Guinea- the tittle is informative and clearly understandable however the focus of your study is on induced abortion particularly unsafe abortion so why did you used the general term ‘’pregnancy termination’’?

2. What do you think about the non-response rate which is very high in this study so how did you manage it?

3. On the conclusion part you said the prevalence of pregnancy termination among married women remains high in PNG. What was your reference to say it is high?

4. On the data source line number 149 you said that **the extracted data on these women included information on their socio-demographic characteristics and the history of termination of pregnancy within the three years preceding the survey.** You did not exclude those mothers without the history of pregnancy within the three years. I raised this issue because it has no importance of including them in this study since they were not pregnant within the three years.

5. The line umber 159 ….**Women were asked whether they had ever terminated a pregnancy. The response was coded as “0” for “No” and “1” for “Yes” for pregnancy termination. For this study, the terms pregnancy termination and induced abortion are used interchangeably**. This part should be written in the operational definition.
